# Asymptomatic Orthopoxvirus Circulation in Humans in the Wake of a Monkeypox Outbreak among Chimpanzees in Cameroon

**DOI:** 10.4269/ajtmh.19-0467

**Published:** 2019-11-25

**Authors:** Sarah Anne J. Guagliardo, Benjamin Monroe, Christian Moundjoa, Ateba Athanase, Gordon Okpu, Jillybeth Burgado, Michael B. Townsend, Panayampalli S. Satheshkumar, Scott Epperson, Jeffrey B. Doty, Mary G. Reynolds, Elisabeth Dibongue, Georges Alain Etoundi, Els Mathieu, Andrea M. McCollum

**Affiliations:** 1 Epidemic Intelligence Service, U.S. Centers for Disease Control and Prevention, Atlanta, Georgia;; 2 Poxvirus and Rabies Branch, U.S. Centers for Disease Control and Prevention, Atlanta, Georgia;; 3 Ministry of Livestock, Fisheries, and Animal Industries, Yaoundé, Cameroon;; 4 Field Epidemiology Training Program, U.S. Centers for Disease Control and Prevention Cameroon Office, Yaoundé, Cameroon;; 5 National Zoonoses Program, Ministry of Health, Yaoundé, Cameroon;; 6 U.S. Centers for Disease Control and Prevention Cameroon Office, Yaoundé, Cameroon;; 7 Hubert Humphrey Global Health Fellowship Program, U.S. Centers for Disease Control and Prevention, Atlanta, Georgia;; 8 Disease Control, Ministry of Health, Yaoundé, Cameroon

## Abstract

*Monkeypox virus* is a zoonotic *Orthopoxvirus* (OPXV) that causes smallpox-like illness in humans. In Cameroon, human monkeypox cases were confirmed in 2018, and outbreaks in captive chimpanzees occurred in 2014 and 2016. We investigated the OPXV serological status among staff at a primate sanctuary (where the 2016 chimpanzee outbreak occurred) and residents from nearby villages, and describe contact with possible monkeypox reservoirs. We focused specifically on Gambian rats (*Cricetomys* spp.) because they are recognized possible reservoirs and because contact with Gambian rats was common enough to render sufficient statistical power. We collected one 5-mL whole blood specimen from each participant to perform a generic anti-OPXV ELISA test for IgG and IgM antibodies and administered a questionnaire about prior symptoms of monkeypox-like illness and contact with possible reservoirs. Our results showed evidence of OPXV exposures (IgG positive, 6.3%; IgM positive, 1.6%) among some of those too young to have received smallpox vaccination (born after 1980, *n* = 63). No participants reported prior symptoms consistent with monkeypox. After adjusting for education level, participants who frequently visited the forest were more likely to have recently eaten Gambian rats (OR: 3.36, 95% CI: 1.91–5.92, *P* < 0.001) and primate sanctuary staff were less likely to have touched or sold Gambian rats (OR: 0.23, 95% CI: 0.19–0.28, *P* < 0.001). The asymptomatic or undetected circulation of OPXVs in humans in Cameroon is likely, and contact with monkeypox reservoirs is common, raising the need for continued surveillance for human and animal disease.

## INTRODUCTION

*Monkeypox virus* (MPXV) belongs to the genus *Orthopoxvirus* (OPXV), which also includes *Variola*, *Cowpox*, and *Vaccinia viruses*. Orthopoxviruses provide immunological cross-protection such that infection with one OPXV or immunization with *Vaccinia virus* (via smallpox vaccination) provides some degree of protection against the others in the genus.^1,2^ Since the eradication of smallpox in 1980, there have been increasing OPXV outbreaks among both animals and humans, suggesting a shift in the ecology and evolution of OPXVs simultaneous with diminishing smallpox vaccine–derived immunity.^3^ *Monkeypox virus* is endemic in West and Central Africa, and human infections are more frequently recognized.^4^ In its most severe form, the clinical presentation of monkeypox is similar to that of smallpox, causing rash following a prodromal period of fever, malaise, headache, and lymphadenopathy.^5–7^ The gravity of monkeypox disease is determined by the exposure route, the strain and dose of the infecting virus, and the baseline health status of the patient. Of the two viral clades, the Congo Basin clade is thought to have more severe disease presentation than the West African clade.^8,9^

**Figure 1.**
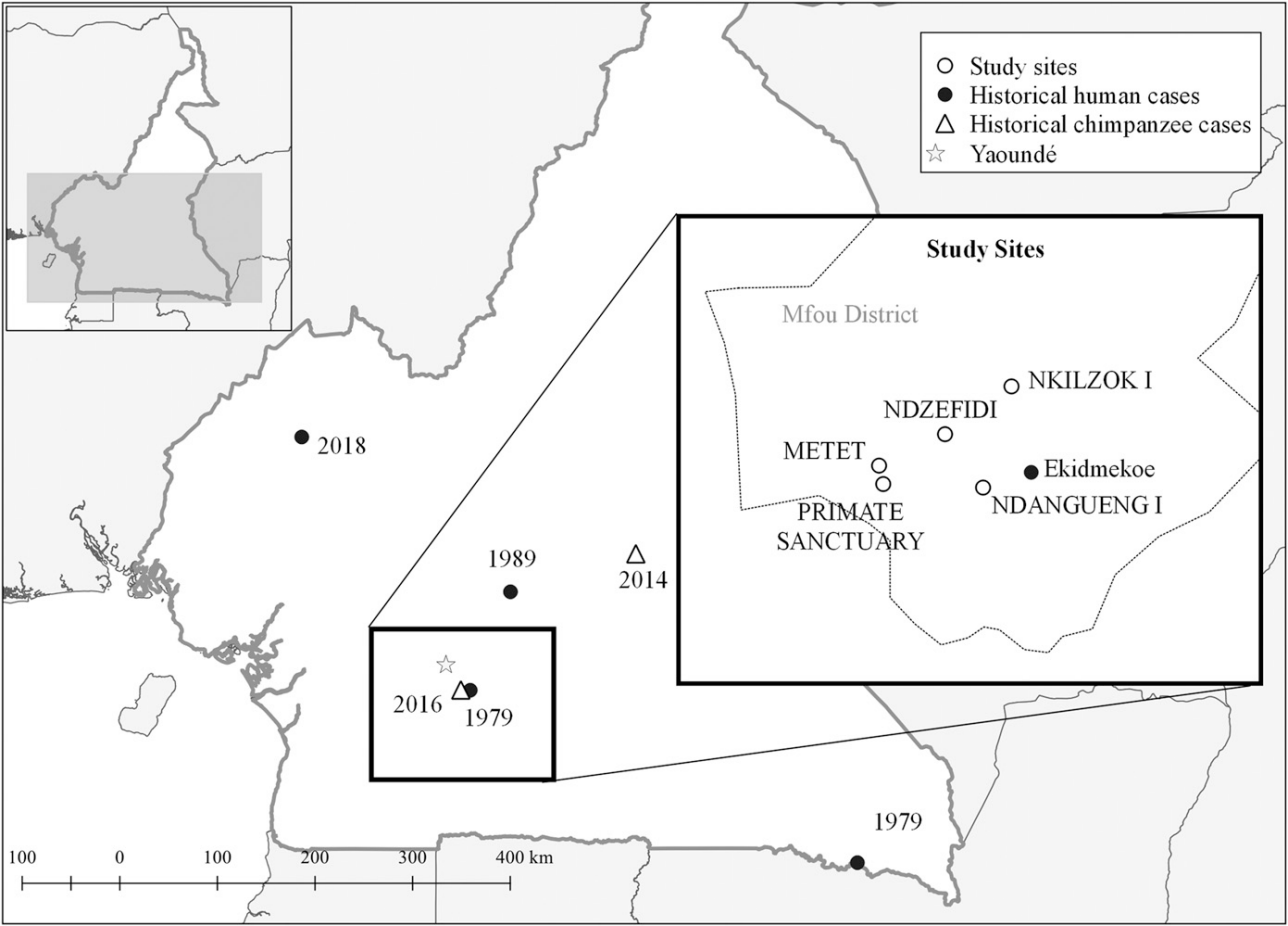
Map of study sites and historically confirmed human and chimpanzee monkeypox cases. Confirmed human and chimpanzee monkeypox cases in Cameroon are shown, with the year of confirmation noted. Human monkeypox cases were last reported in Mfou district in 1979 in Ekidmekoe village, and an outbreak of monkeypox occurred in captive chimpanzees in 2016. Participants were recruited from the villages of Metet, Nzdefidi, Ndangueng I, and Nkilzok I. Employees of the nearby primate sanctuary were also invited to participate.

Monkeypox is on the rise in West and Central Africa, and in Cameroon, there have been a number of epidemiologic and epizootic events related to this emerging zoonosis.^4^ Historical human cases have been noted in 1979 in Ekidmekoe village (Mfou district), in 1980 in the city of Moloundou, and in 1989 in Nkoteng village (Figure 1).^10–13^ More recently, in April and May of 2018, there were reports of human monkeypox in northwest and southwest regions, with one confirmed and 15 suspected cases.^14,15^ Monkeypox outbreaks have also occurred among captive chimpanzees housed at wildlife sanctuaries in Sanaga-Yong in Sanaga-Yong in 2014 and in Mfou district in 2016.^16^ Although workers were likely exposed to the virus while caring for sick animals during the recent chimpanzee outbreaks, questions remain regarding the prevalence of subclinical disease and circulation and exposure to OPXVs in the area.

The reservoir(s) of MPXV remain(s) a mystery, but current data suggest small mammals (i.e., rodents) are involved in sylvatic circulation and maintenance of the virus^17^ and subsequent introduction to human populations. Monkeypox virus has only been isolated twice from wild animals: once from a rope squirrel (*Funisciurus anerythrus*) in the Democratic Republic of Congo (DRC) in 1985 and once from a sooty mangabey (*Cercocebus atys*) in Côte d’Ivoire in 2012.^18,19^ Ecologic investigations from Ghana and DRC showed that animals most likely to have anti-OPXV antibodies included those of the genera *Graphiurus* (dormice), *Cricetomys* (giant pouched rats), *Funisciurus* (rope squirrels), and *Heliosciurus* (sun squirrels). *Lemniscomys* (striped mouse) and *Tatera* (gerbil) have also been implicated, in addition to *Oenomys hypoxanthus* (rufous-nosed rat) and *Petrodromus tetradactylus* (elephant shrew).^17,20^ Gambian rats (*Cricetomys* spp.) in particular have been repeatedly associated with MPXV, either through detection of anti-OPXV antibodies or MPXV DNA.^17,20–22^ Falendysz et al. (2015) describe Gambian rats as possessing characteristics of a “typical” reservoir—that is, they have the ability to amplify and transmit virus, without the appearance of serious disease.^23^ In West and Central Africa, hunting, selling, and preparing of wild game/“bushmeat” are commonplace,^24^ and contact with these wild animal reservoirs through hunting and preparation of meat is presumed to be a risk factor for monkeypox infection.^21^ Transmission to humans occurs through contact with infected bodily fluids, such as blood, salivary/respiratory droplets, and lesion exudates and crusts.^25^

The purpose of this report was to describe the seroprevalence of OPXVs in humans in Mfou district, Cameroon, and to explore the frequency and type of contact with bushmeat, with focus on Gambian rats. An improved understanding of transmission of OPXVs, the extent of subclinical disease, and risk factors associated with infection will inform the local prevention and response to this serious public health threat.

## METHODS

### Study area.

The investigation was conducted in Mfou district, in the central region of Cameroon, and was carried out at a primate sanctuary, in addition to four nearby villages: Metet, Nzdefidi, Ndangueng I, and Nkilzok I. This region was selected as the site for the investigation because it is where the monkeypox outbreak occurred in captive chimpanzees in 2016. The most recent national census in 2011 estimated the Mfou district population to be 71,373 individuals, the majority of whom are employed as traders and farmers.^26^ The capital of Mfou district is a town by the same name, located about 25 km south of Yaoundé (Figure 1).

### Study design.

This investigation used a cross-sectional design with two cohorts including staff employed at the primate sanctuary and residents from the four villages mentioned earlier. The study was carried out in October 2017, approximately 1 year after the chimpanzee outbreak (in August–September 2016). On arrival in each of the five study locations, park staff or community members attended an educational session about monkeypox. Afterward, adults aged ≥ 18 years were invited to participate in the project which consisted of a questionnaire and blood sample collection. All communication was conducted in French, English, or local languages, as necessary. Participants were compensated for their participation and, at any time, could choose to stop the questionnaire or blood draw.

This investigation was determined to be public health non-research by the delegated authority at CDC’s National Center for Zoonotic and Infectious Diseases (protocol number 100417SG). The investigation protocol was approved by the Cameroonian Ministry of Health, in addition to a Cameroonian Institutional Review Board. Written informed consent was acquired from each participant.

### Questionnaire.

Questionnaires consisted of paper surveys administered in French or a local language by representatives of the Ministry of Health. Information was collected on participant demographics, socioeconomic status, education, and contact with wildlife. Participants were shown photos of animals that are possible MPXV hosts and were asked whether they had contact with these animals within 10 months before the survey administration (the time from January 2017 through October 2017). We also asked participants about the type of contact they experienced with each species, such as hunting/trapping, selling/touching, or preparing meat. Furthermore, participants were shown a photo of typical lesions from monkeypox patients and were asked if they recalled having these lesions themselves or having contact with anyone with similar lesions. Additional questions specific to park staff were asked about contact with sick chimpanzees during the 2016 outbreak and use of personal protective equipment.

### Specimen collection and laboratory analyses.

One 5- to 10-mL whole blood specimen was collected from each participant via sterile venipuncture technique by a trained phlebotomist. Laboratory specimens were labeled with the date and a unique participant identifier that corresponded to the questionnaire. The blood specimens were stored at 4°C and transported to the district hospital where the serum was separated by centrifugation, aliquoted, and stored at 4°C. The specimens were transferred to Yaoundé and were stored at −20°C before and during shipment to the CDC’s Poxvirus Laboratory in Atlanta, Georgia, for analysis to detect the presence of anti-OPXV antibodies. Orthopoxvirus IgM and IgG antibodies in patient sera were detected by ELISAs at 1:50 and 1:100 dilutions, as previously described.^27^ This assay is OPXV-generic and, therefore, cannot be used to distinguish MPXV antibodies from other OPXV antibodies.

### Data management and analysis.

Questionnaire data were recorded on paper forms, entered into a Microsoft Access database, and were later merged with laboratory data by a unique participant identifier. Data analyses were conducted in SAS, version 9.4 (SAS Institute, Inc., Cary, NC).

We used five outcome variables that corresponded to different types of contact with Gambian rats, including any contact, trapping/hunting, selling/touching, preparing, or eating. Although there are several candidate reservoirs for MPXV, we focused on contact with Gambian rats (*Cricetomys* spp.) because both laboratory and field studies have repeatedly implicated this species as a possible reservoir^20–22,23,28^ and because contact with this species was common enough to render sufficient statistical power (> 50% of study participants reporting contact). Analysis of associations between seropositivity and survey outcomes was not possible because of the low numbers of seropositive individuals.

We characterized relationships between the five outcome variables and demographic, socioeconomic, and educational variables (chi-square/Fisher’s exact test). Variables that approached statistical significance (*P* < 0.15) in the bivariate analyses were input into univariable logistic regression models, using the cohort (sanctuary staff and community) as the clustering variable in a repeated measures term in *proc genmod* in SAS. Selected predictor variables (*P* < 0.10 in univariable models) were then included in multivariable models, using a backward selection stepwise procedure to identify relevant predictors (*P* < 0.05). When two similar variables were both significant in the univariable modeling step, the model with the best fit (i.e., Akaike information criterion) was selected for further analysis. For multivariable models, a backward stepwise procedure was used to determine the best model in *proc hpgenselect*, with an entry criterion of *P* = 0.10 and a selection criterion of *P* = 0.08. Model assumptions were evaluated using collinearity diagnostics; a condition index less than 15 was presumed to reflect independence of predictor variables.

## RESULTS

### Study population.

Participants included 45 employees from the primate sanctuary and 80 participants from the four nearby villages. Participant ages ranged from 18 to 83 years (median: 37 years); 50.8% were born after routine smallpox vaccination ceased in 1980 (maximum of 37 years old). The average household size was 5.82 individuals. In comparison with community members, park employees were more likely to be male (chi-square = 6.7, *P* < 0.01), younger (*P* < 0.001), more educated (*P* < 0.01), and reported less frequent forest visits (*P* < 0.001) (Table 1).

**Table 1:** Characteristics of the study population

Select variable	*n* (%)	*n* (%)	*n* (%)	χ^2^	*P*-value
Gender					
Male	81 (64.8)	38 (84.4)	43 (53.8)	**6.66**	**0.0098**
Female	44 (35.2)	7 (15.6)	37 (46.3)		
No response	0	–	–		
Smallpox vaccination*					
No	63 (50.4)	31 (68.9)	32 (40.5)	**6.38**	**0.012**
Yes	61	14 (31.1)	47 (59.5)		
No response	1	–	–		
Age-group (years)					
18–29	36 (29.0)	17 (37.8)	19 (24.1)	NA	**< 0.0001**†
30–39	31 (25)	16 (35.6)	15 (19.0)		
40–49	23 (18.5)	10 (22.2)	13 (16.5)		
≥ 50	34 (27.4)	2 (4.4)	32 (40.5)		
No response	1	–	–		
Education completed					
None	6 (4.8)	1 (2.2)	5 (6.3)	NA	**0.0064**†
Some primary	46 (36.8)	10 (22.2)	36 (45)		
Some secondary	60 (48)	25 (55.6)	35 (43.8)		
Superior	13 (10.4)	9 (20)	4 (5)		
No response	0	–	–		
Forest visits					
< Once per week	15 (12)	10 (22.2)	5 (6.3)	NA	**0.0006**†
> Once per week	104 (83.2)	30 (66.7)	74 (92.5)		
Never	6 (4.8)	5 (11.1)	1 (1.3)		
No response	0	–	–		

Bold denotes statistical significance. In total, 125 individuals participated in the investigation, including 45 primate sanctuary employees and 80 community members. Significant demographic differences were observed between these two cohorts.

* Routine smallpox vaccinations in Africa ceased in ∼1980. Persons aged less than or equal to 37 years at the time of enrollment in this study were not expected to have had the opportunity for childhood vaccination to protect against smallpox.

† Fisher’s exact test, *P*-value.

The most common occupations among the 80 community members included farming (only farming, 47, 58.8%), homemakers (10, 12.5%), farming and some other occupation (8, 10.0%), tradesmen (7, 8.8%), and students/teachers (7, 8.8%). Primate sanctuary staff (*n* = 45) comprised animal caregivers, such as veterinarians and keepers (27, 60%), and other types of workers including masons, maintenance staff, and security guards (18, 40%).

### Serological results.

The proportion of individuals who were IgG positive for anti-OPXV antibodies was 34.4% (43/125). Among the 63 participants less than the age of smallpox vaccination cutoff, four (6.3%) were IgG positive (Table 2). Notably, however, one of these participants had a very low IgG value of 0.02. One participant from the village of Metet tested IgM positive (and IgG positive) for anti-OPXV antibodies, indicating a recent OPXV exposure (1/63, 1.6%). This individual had reported frequent venturing into the forest (> once per week) and also reported encountering Gambian rats and sun squirrels during the period of January through October 2017. Specific types of contact with both animals included selling/touching animals with their bare hands (alive or dead), preparing for cooking, eating, and having contact with urine or feces.

**Table 2:** Characteristics of IgG-seropositive individuals under vaccination age

Participant	Gender	Location	IgG value	IgM value	Occupation	Forest visits
1	Female	Metet	**0.83**	**0.12**	Farmer	> Once per week
2	Male	Ndzefidi	**0.49**	−0.04	Farmer and student	> Once per week
3	Male	Primate sanctuary	**0.20**	−0.05	Primate sanctuary (animal care)	> Once per week
4	Male	Ndzefidi	**0.02**	−0.11	Student	> Once per week

Positive IgG and IgM values (in bold) indicate a past or recent exposure to an orthopoxvirus, respectively. One participant (#1) tested positive for both anti-orthopoxvirus IgG and IgM antibodies. Note that participant #4 had a very low positive IgG value, which is considered to be an equivocal result.

The four IgG-seropositive individuals included one employee of the primate sanctuary (animal caregiver) and three community members who were a farmer, a farmer/student, and a student. No participants reported a history of smallpox-like disease or rash illness resembling monkeypox, and the animal handler did not take care of the sick chimpanzees during the 2016 outbreak. Among the seven participants who touched the sick chimpanzees during the outbreak, five were IgG negative. The remaining two persons who were IgG positive were born before 1980 and, therefore, had likely received the smallpox vaccine.

### Analysis of animal exposures.

Contact with the following animals was reported among participants: porcupines (82, 65.6%), Gambian rats (71, 56.8%), sun squirrels (35, 28%), and rope squirrels (33, 26.4%). Although study participants reported encounters with a diversity of animals, our analysis specifically focused on modeling contact with Gambian rats.

**Figure 2.**
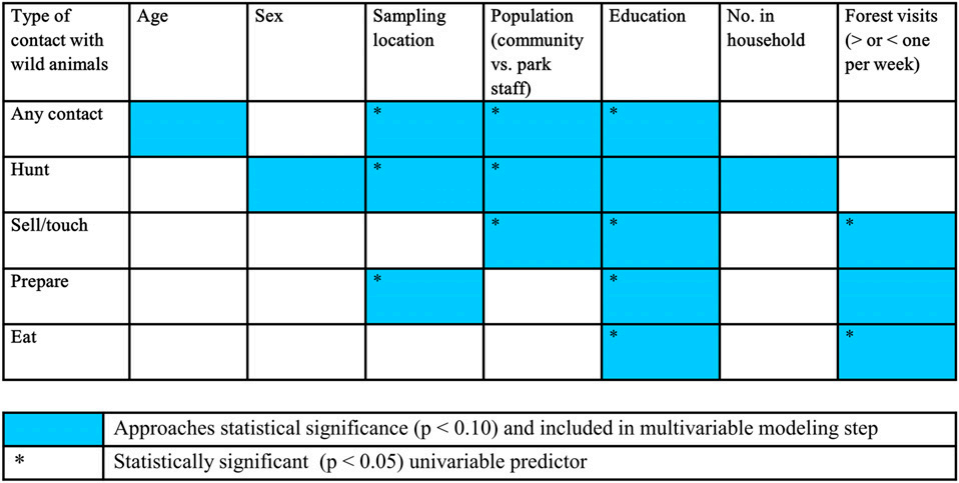
Summary of univariable modeling results. The sampling location refers to the location from which the participant was recruited, either the primate sanctuary or one of the four villages included in the study. The level of education refers to whether the participant had no education or a primary, secondary, or superior level of education. This figure appears in color at www.ajtmh.org.

Variables that were considered in the univariable logistic regression models predicting different modes of contact with Gambian rats included age, gender, sampling location (village), population (community versus park staff), education completed, household size, and forest visits. Univariable regression models (Supplemental Table 1) showed that participants with no formal education were significantly more likely to report any type of contact with Gambian rats, in addition to selling/touching, preparing, and eating (Figure 2). Park staff members were less likely to engage in hunting, selling/touching, or having any type of contact with Gambian rats.

Multivariable modeling showed that when adjusting for the level of education, participants reporting more than one visit to the forest per week were 3.36 times as likely as those reporting < 1 visit to the forest per week to have recently eaten Gambian rats (95% CI: 1.91–5.92, *P* < 0.001) (Table 3). In comparison with community members, primate sanctuary staff were 0.23 times as likely (95% CI: 0.19–0.28, *P* < 0.001) to have recently touched or sold Gambian rats, when accounting for the level of education. All model assumptions were satisfied.

**Table 3:** Multivariable models predicting contact with Gambian rats (ate and sold/touched)

Outcome	Variable (ref)*	aOR	(95% CI)	SE	*P-*value
Ate	Education (none)				
	Some primary	1.45	(0.70–2.99)	0.81	0.7256
	Some secondary	1.12	(0.37–3.46)	0.79	0.9698
	Superior	0.05	(0.66–0.05)	0.75	**0.0152**
	Forest visits (< once per week)				
	> Once per week	3.36	(1.91–5.92)	0.29	**< 0.0001**
Sold/touched	Population (community)†				
	Park	0.23	(0.19–0.28)	0.29	**< 0.0001**
	Education (none)				
	Some primary	1.01	(0.66–1.55)	0.36	0.0440
	Some secondary	1.37	(1.01–1.86)	0.34	**0.0024**
	Superior	0.20	(0.06–0.62)	0.65	0.1594

Bold denotes statistical significance. After adjusting for the level of education in both models, we found that people visiting the forest > 1 time per week were 3.4 times as likely to have recently eaten Gambian rats. Being an employee of the primate sanctuary was protective against selling/touching Gambian rats.

* Reference categories are reported in parentheses.

† The variable “population” refers to whether the participant was a community member or an employee of the primate sanctuary (indicated by “park”).

## DISCUSSION

We identified the presence of anti-OPXV antibodies in participants less than the age of smallpox vaccination in Mfou district, Cameroon, suggesting undetected or possible asymptomatic circulation of an OPXV in human populations in this region. Although the ELISA used in this study is a generic OPXV test, we postulate that MPXV is the most likely culprit because of the historical cases in humans and in chimpanzees in Mfou, Cameroon,^10,16^ and because it is the only OPXV reported in humans in Africa. Even so, it is still possible that the anti-OPXV antibodies detected here are the result of exposures from a different, possibly unknown/cryptic OPXV. Taterapox virus, for example, is another (remote) possibility—but it is not known to occur in humans, and some scholars consider it to be “cryptic,” as it is rarely detected even in its presumptive primary (rodent) hosts.^29,30^ When adjusting for the age of smallpox vaccination, the observed IgG seropositivity (6.3% in persons aged ≤ 37 years) was much lower than previous measures in Ghana (37%)^20^ and northern Republic of the Congo (49.1%),^31^ but higher than that reported in Sierra Leone (1.3%).^32^ Notably, one participant was IgM positive, indicating a recent exposure to an OPXV. Despite this evidence for active circulation, very few human cases of monkeypox have been reported in Cameroon. This deficit between serological results and reported cases may be attributable to limited awareness among health care providers and insensitive surveillance systems, possibly leading to either overlooking less severe cases or misidentification of monkeypox (i.e., confusion with varicella).

Our analysis of animal exposures showed notable differences in animal contact patterns among park staff and people from surrounding towns. This could be attributable to differences in formal education, attitudes about conservation, and/or socioeconomic status. For example, the park has a strong tradition of conservation, likely discouraging its employees from hunting or poaching wild animals. A higher level of education was associated with not eating Gambian rats, and selling or touching Gambian rats was more likely to occur among villagers with an intermediate level of education. This could be because merchants selling wild game may have some degree of formal education, or alternatively, individuals with lower levels of education may be poorer and, therefore, more likely to consume wild game they have hunted. Other works have shown a positive correlation between bushmeat consumption and wealth,^33^ but the relationship between socioeconomic status and bushmeat consumption is complex —in the context of an Ebola outbreak, for example, bushmeat consumption among low-income households decreased significantly more than in high-income households.^34^ Further investigation is required to more precisely describe the relationships between socioeconomic status and behaviors surrounding the consumption of wild game in Cameroon.

Among villagers, we found contact with wildlife to be common, with near ubiquitous contact with porcupines. In addition to reports of hunting porcupines noted in our survey data, which reflects the popularity of this protein source, we also anecdotally observed the abundance of porcupine meat in local restaurants. Research from Nigeria,^35^ Gabon,^36^ and the DRC^21^ have similarly identified porcupines as a common food source. As other researchers have noted,^21^ serological surveys of porcupines could be used to determine whether they may serve as a potential reservoir of MPXV and other zoonoses.

Some limitations of our analysis of animal exposures should be noted. First, as in other OPXV serosurveys,^20,31,32^ we used age as a proxy for receipt of smallpox vaccination. None of the participants under the age cutoff (≤ 37 years) reported a history of smallpox vaccination, but it is still possible that participants misreported their age. Our population consisted of a targeted convenience sample—only individuals who were present in communities on arrival of study staff were able to participate. It is therefore possible that our sample of participants is not representative of the population in this region. Other sources of possible bias might include social desirability (when answering questions about contact with sick animals), self-selection bias (animal care staff may be more inclined to participate), and recall bias (because exposures to the sick animals occurred many months ago).

Despite these limitations, our results suggest that although unrecognized, MPXV (or OPXVs in general) may circulate in humans and wild mammals in central Cameroon. Human monkeypox warrants attention from the public health and scientific communities because of its grave clinical manifestations and potential for international spread, as exemplified by recent importation of cases from Nigeria to the United Kingdom, Israel, and Singapore.^37–39^ As cases continue to rise, it will be important for public health entities to improve surveillance systems through clinical trainings and community-wide education efforts (Indeed, before this investigation, we led a comprehensive training for 33 health care workers in the region). Future public health responses to monkeypox could strengthen monkeypox prevention efforts by enhancing training in clinical case recognition, case management, infection prevention and control, appropriate use of personal protective equipment, and epidemiological reporting.^40^

## Supplemental table

Supplemental materials
