# Supplementary material for: Asymptomatic Orthopoxvirus Circulation in Humans in the Wake of a Monkeypox Outbreak among Chimpanzees in Cameroon
Source: Am J Trop Med Hyg. 2019 Nov 25;102(1):206–12. doi: 10.4269/ajtmh.19-0467 (PMC6947779; doi:10.4269/ajtmh.19-0467)
Supplement: Supplementary file 1 [file tpmd190467.SD1.pdf]

Supplemental Table S1. Univariable models predicting contact with Gambian rats.

Outcome	Variable (ref)§	OR	(95% CI)		SE	P	QICu/ AIC
Any contact	Age*	0.50	(0.50-	0.51)	0.010	0.056	166.83†
	Location(Park)*						140.62
	Metet	2.80	(1.01-	7.77)	0.62	**0.0017**	
	Ndangueng I	1.038E11	(0-	Infty)	97120.96	0.99	
	Ndzefidi	2.63	(1.16-	5.93)	0.53	**0.0004**	
	Nkilzok I	1.43	(12.63-	1.43)	0.65	**0.0003**	
	Education(None)*						164.75†
	Some primary	0.46	(0.07-	3.11)	0.35	0.42	
	Some secondary	0.30	(0.06-	1.54)	0.55	0.15	
	Superior	0.06	(0.01-	0.34)	0.59	**0.0014**	
	Forest visits(<Once)						166.10†
	>Once per week	1.89	(0.77-	4.65)	0.46	<0.0001	
	Pop.(Community)*‡						
	Park	0.41	(0.41-	0.41)	0.24	**<0.0001**	140.86
Hunted	Sex (Female)						149.74
	Male	2.15	(0.8-	5.8)	0.51	0.13	94.03
	Location(Park)*						
	Metet	3.79	(1.07-	13.5)	0.65	**0.039**	
	Ndangueng I	29.25	(5.05-	169.47)	0.90	**0.0002**	
	Ndzefidi	8.0	(2.59-	24.74)	0.58	**0.0003**	
	Nkilzok I	5.90	(1.76-	19.88)	0.62	**0.0041**	
	Education(None)*						165.38†
	Some primary	4.20	(0.87-	20.29)	0.80	0.07	
	Some secondary	3.57	(0.62-	20.70)	0.90	0.16	
	Superior	0.42	(0.02-	7.55)	1.48	0.55	
	Num. in HH*	0.51	(0.50-	0.51)	0.01	0.05	
	Forest visits(<Once)						168.10†
	>once per week	2.26	(0.58-	8.71)	0.69	0.24	
	Pop.(Community)*‡						150.04
	Park	0.14	(0.09-	0.22)	0.23	**<0.0001**	
Sold/ Touched							
	Location(Park)						148.38
	Metet	2.17	(0.82-	5.70)	0.60	0.0028	
	Ndangueng I	1.038E11	(0-	Infty)	97120.96	0.99	
	Ndzefidi	1.90	(0.88-	4.09)	0.52	0.0014	
	Nkilzok I	2.50	(0.97-	6.44)	0.59	0.0011	
	Pop.(Community)*‡						150.30†
	Park	0.36	(0.36-	0.36)	0.23	**<0.0001**	
	Education*						168.84†
	Some primary	1.71	(0.95-	3.07)	0.30	0.075	
	Some secondary	1.50	(0.71-	3.19)	0.38	0.29	
	Superior	0.18	(0.05-	0.71)	0.70	**0.014**	
	Forest Visits*						170.22†
	>once per week	3.07	(1.47-	6.41)	0.38	**0.0028**	

Outcome	Variable (ref)§	OR	(95% CI)		SE	P	QICu/ AIC
Prepared							143.73
	Location(Park)*						
	Metet	1.71	(0.67-	4.35)	0.60	0.0026	
	Ndangueng I	1.038E11	(0-	Infty)	97120.96	0.99	
	Ndzefidi	2.22	(1.01-	4.88)	0.54	**0.0001**	
	Nkilzok I	2.50	(0.97-	6.44)	0.60	0.0003	
	Education*						164.92†
	Some primary	1.88	(1.07-	3.28)	0.31	**0.0417**	
	Some secondary	1.31	(0.38-	4.47)	0.42	0.5262	
	Superior	0.08	(0.01-	1.01)	1.23	0.0434	
	Forest visits						171.78†
	>once per week	1.42	(0.61-	3.32)	0.37	0.0048	
Ate							
	Age	0.50	(0.50-	0.51)	0.01	0.21	169.14†
	Education*						167.0†
	Some primary	2.29	(1.15-	4.55)	0.79	0.87	
	Some secondary	0.50	(0.51-	4.43)	0.78	0.71	
	Superior	0.09	(0.95-	0.09)	0.75	**0.011**	
	Forest visits*						167.31†
	>once per week	0.75	(0.64-	0.83)	0.25	**<0.0001**	

Bold denotes statistical significance.

§Reference categories are reported in parentheses.

†QICu

*Included in multivariable modeling step (P<0.10).

‡ The variable ‘population’ refers to whether the participant was a community member or an employee of the primate sanctuary (indicated by ‘park’).
